# Molecular Orientation and Mechanical Properties of Biomass-Derived Aliphatic Polyamide (PA11) by High-Pressure Compression Molding

**DOI:** 10.3390/ma19030513

**Published:** 2026-01-28

**Authors:** Keisuke Ura, Shotaro Nishitsuji, Yutaka Kobayashi, Hiroshi Ito

**Affiliations:** 1 Industrial Technology Institute, Miyagi Prefectural Government, Sendai 981-3206, Japan; 2 Graduate School of Organic Materials Science, Yamagata University, Yonezawa 992-8510, Japan; nishitsuji@yz.yamagata-u.ac.jp; 3 Research Center for GREEN Materials and Advanced Processing, Yamagata University, Yonezawa 992-8510, Japan; kobayashi.y@yz.yamagata-u.ac.jp

**Keywords:** high-pressure compression molding, biomass-derived aliphatic polyamide, molecular orientation

## Abstract

**Highlights:**

**What are the main findings?**

- This study demonstrated that high-pressure thermal compression molding significantly modifies the crystalline structure and mechanical properties of biomass-derived aliphatic polyamide PA11, specifically the grade Rilsan^®^ BMN O TLD manufactured by Arkema—a fully bio-based resin derived from castor oil.
- The tensile fracture strength reached a maximum—approximately 2.4 times higher than that of the uncompressed sample—under experimental conditions of 140 °C and 1000 kN. This enhancement is proposed to be primarily attributable to the molecular orientation and crystallization of the δ’, which remained stable without undergoing Brill transition even after cooling to room temperature, while its crystallinity increased, as supported by POM observations, WAXS analysis, and DSC analysis. In contrast, at 180 °C, although the degree of crystallinity increased, molecular orientation decreased, resulting in reduced tensile strength.
- These findings indicate that the mechanical properties of a fully biomass-derived aliphatic polyamide, PA11, which exhibits crystal polymorphism, are governed by a complex interplay among phase transitions, molecular orientation, and crystallization, all of which are strongly influenced by temperature and pressure conditions.

**What are the implications of the main findings?**

- If the mechanical properties of the biomass-derived polyamide PA11 can be enhanced through high-pressure thermal compression molding, it could be utilized in a wider range of applications. This suggests the potential to shift from petroleum-derived polymers to renewable, biomass-derived polymers, thereby contributing to environmental sustainability.
- These results suggest that, even for polymers whose crystalline phases or structures typically change upon cooling to room temperature, the method has the potential to suppress such phase transformations. This capability may improve durability against strength degradation and dimensional changes under service conditions, and we therefore consider it a topic for future study.
- Expanding the application of this method to other biomass-derived polymers with various crystal polymorphs may allow temperature and pressure conditions to be combined to control phase transitions, molecular orientation, and crystallization. This approach has the potential to contribute not only to improved mechanical strength but also to enhancements in a range of other material properties.

**Abstract:**

This study investigates the effects of high-pressure compression molding on the molecular orientation and mechanical properties of biomass-derived aliphatic polyamide (PA11). Tensile fracture strength exhibited a significant increase—up to 2.4 times that of untreated samples—under conditions of 1000 kN and 140 °C. Differential Scanning Calorimetry (DSC) and Wide-Angle X-ray Scattering (WAXS) analyses revealed a temperature- and pressure-dependent shift in crystalline phases, suggesting a transition from α’ to phase. The δ’ phase, formed by high-pressure compression molding, is retained even after cooling to room temperature (i.e., Brill transition was not observed). In addition, polarized optical microscopy (POM) observations further supported the presence of changes in molecular orientation. This enhancement (under conditions of 1000 kN and 140 °C) is primarily attributed to the molecular orientation. However, it is also noteworthy that the formation of the δ’ phase is accompanied by an increase in the degree of crystallinity, and that this δ’ phase is retained even after cooling to room temperature without undergoing a Brill transition. In contrast, at 180 °C, although the degree of crystallinity increased, molecular orientation decreased, resulting in reduced tensile strength. These findings indicate that the mechanical properties of PA11 are governed by a complex interplay among phase transitions, molecular orientation, and crystallization, all of which are strongly influenced by temperature and pressure conditions. These findings demonstrate that high-pressure compression molding is an effective method for enhancing the mechanical properties of PA11 through controlled phase transition and orientation

## 1. Introduction

In recent years, growing environmental awareness has led to efforts across various fields to conserve fossil resources and reduce greenhouse gas emissions. In the field of polymer materials, biomass-derived plastics have attracted considerable attention from the perspective of carbon neutrality. As they play a crucial role in both reducing environmental impact and promoting resource circulation, biomass-derived plastics have been increasing in presence year by year [1,2].

The castor bean (*Ricinus communis*), native to Africa and belonging to the Euphorbiaceae family, grows as a perennial plant in tropical and subtropical regions, and as an annual plant in temperate regions. Its seeds yield castor oil, from which 11-aminoundecanoic acid is derived. This compound undergoes polycondensation to form aliphatic polyamides. PA11, a biomass-derived aliphatic polyamide made from castor oil, is characterized by its carbon content being entirely sourced from renewable plant-based resources (i.e., 100% bio-based). It is an engineering plastic with a melting point of approximately 187 °C, offering excellent mechanical properties, chemical resistance, low water absorption, and ferroelectricity [3,4,5].

Like other aliphatic polyamides such as PA6 (polyamide 6), PA11 is known to exhibit polymorphic crystallinity and undergo a Brill transition, in which its crystal structure changes depending on temperature [6,7,8,9,10,11,12,13,14,15,16,17,18,19,20,21,22,23,24,25,26,27,28,29,30,31,32]. Therefore, this transition is considered particularly important when optimizing PA11 for high-performance applications, as differences in molding methods may influence its physical properties, including crystallinity, tensile strength, and dimensional stability. Currently, PA11-based products are widely used across various sectors, including the automotive, chemical, and sporting goods industries, and are applied in components such as fuel lines in commercial vehicles, air brake tubing, gas piping, badminton shuttlecocks, and dishwasher baskets [3,4,5]. It is considered that further improvements in the physical properties of PA11, such as mechanical strength, could expand its range of applications and potentially contribute to reducing environmental impact across a wide range of industries.

In the field of metallurgy, numerous studies have been conducted to enhance the mechanical properties—such as strength and toughness—of metals through forging and rolling processes. These techniques, performed at temperatures below the melting point, involve striking the metal forcefully with tools such as hammers or presses, and shaping it using dies or rolling mills. Such deformation processes refine and orient the internal crystal structure of the metal [33,34,35].

In the field of plastics, improvements in physical properties through forging or rolling have been reported for materials such as PP (polypropylene), PLA (polylactic acid), PTFE (polytetrafluoroethylene), and polymethacrylate. During compression molding, materials undergo thinning and elongation as a result of the applied load or the rolling ratio in roll pressing. In uniaxial compression molding, biaxial stretching and molecular orientation are induced. These compression molding techniques have been reported to significantly affect molecular orientation, crystallinity, and microstructural development, thereby altering the resulting physical properties of the material [36,37,38,39,40,41,42,43,44]. However, several studies have reported improvements in the properties of PA11, research that correlates mechanical properties with changes in molecular orientation and crystalline phases using these compression molding techniques remains limited to date.

Enhancement of mechanical properties through these compression molding techniques would increase the versatility of PA11 (biomass-derived polyamide), enabling its application beyond conventional products to a broader range of uses, and potentially contributing to the realization of a circular society. Accordingly, this study investigates the effects of high-pressure thermal compression molding on the mechanical properties, molecular orientation, and crystalline structure of bio-based aliphatic polyamide (PA11). Although changes in the physical properties of PA11 resulting from various molding techniques have been reported, the stabilization behavior of the δ ‘phase during compression molding and the potential for pressure-induced orientation—such as uniaxial or biaxial—have not been sufficiently investigated.

In particular, quantitative insights into how pressure and temperature conditions during molding influence crystalline phase transitions, molecular orientation, and mechanical properties remain limited.

We hypothesize that the high-pressure compression molding technique can affect the crystalline phase transitions and orientation of polymorphic PA11 through pressure and temperature conditions, thereby enhancing the mechanical properties. This study was conducted to elucidate this effect.

## 2. Materials and Methods

### 2.1. Materials and Preparation

**Figure 1:**
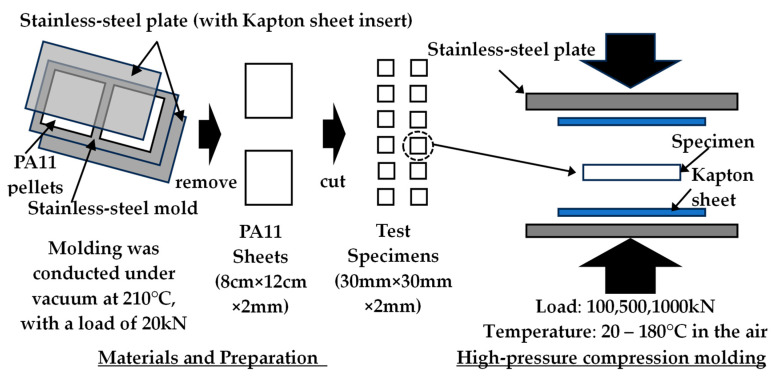
Schematic of specimen preparation and high-pressure compression molding.

In this study, commercially available PA11 (Rilsan^®^ BMN O TLD, a fully bio-based resin derived from castor oil and manufactured by Arkema, La Défense, France; Density: 1.03 × 10^3^ kg/m^3^; MFR: 21.6 g/10 min (2.16 kg/210 °C, [45])) was used. Prior to use, the resin pellets were dried under vacuum at 80 °C for 16 h. To prepare test specimens, PA11 pellets were molded using a stainless-steel mold (outer dimensions of 15 cm × 20 cm × 2 mm thickness, and two areas with inner dimensions of 12 cm × 8 cm × 2 mm thickness) and a vacuum hot press machine (IMC-11FA, manufactured by Imoto Machinery Co., Ltd., Kyoto, Japan). Molding was conducted under vacuum at a mold temperature of 210 °C using the hot press method, with a holding time of 10 min. After removal from the press, the molded material was cooled by sandwiching it between metal plates maintained at 20 °C. The resulting PA11 sheet (2 mm thick) was then cut using a machining tool to produce test specimens measuring 30 mm in length, 30 mm in width, and 2 mm in thickness (Figure 1).

### 2.2. High-Pressure Compression Molding Method

Test specimens were prepared by high-pressure compression molding using a hydraulic hot press (SVP10, Satoh Machinery Works Co., Ltd., Nagoya, Japan) with a maximum load capacity of 1000 kN. The specimens were sandwiched between two metal plates, with Kapton sheets coated with a lubricant (GA-9750M, Daikin Industries, Ltd., Osaka, Japan) placed on both sides. Compression molding was conducted under loads ranging from 100 to 1000 kN at temperatures between 20 and 180 °C, with a preheating time of 10 min and a pressing time of 1 min. To facilitate the future applicability of this method, it was necessary to select a temperature range suitable for practical processing. Accordingly, the lower temperature limit was set near room temperature (20 °C), while the upper limit was defined as 180 °C, which is below the primary melting peak, to avoid the influence of melting. Furthermore, to elucidate the effect of pressure on physical properties and related characteristics, the applied load was varied from 100 kN (250 MPa) to 1000 kN (2500 MPa), corresponding to a range from typical injection molding pressures to tenfold that level. After removal from the press, the molded material was cooled between metal plates maintained at 20 °C for 5 min (Figure 1).

### 2.3. Differential Scanning Calorimetry (DSC)

Differential scanning calorimetry (DSC) was employed to investigate the melting point and melting behavior of high-pressure thermal compressed PA11 specimens, using an X-DSC 7000 instrument (Hitachi High-Tech Analysis Corporation, Minato-ku, Tokyo, Japan). The heating rate was set to 3 °C/min, with a measurement temperature range of 20–220 °C. Approximately 10 mg of each sample was used, and the measurements were conducted under a nitrogen atmosphere with a gas flow rate of 40 mL/min. The degree of crystallinity, X (%), was calculated using the following equation:

X (%) = ΔH_m_/ΔH^0^_m_ × 100 (1)

where ΔH_m_ and (ΔH^0^_m_ = 226 J/g) [12] are the fusion enthalpies of the sample and of the completely crystalline PA11, respectively.

### 2.4. Wide-Angle X-Ray Scattering (WAXS)

Wide-angle X-ray scattering (WAXS) was employed to evaluate changes in the crystalline structure and orientation of PA11 specimens subjected to high-pressure thermal compression molding, using an automated multipurpose X-ray diffractometer (SmartLab, Rigaku Holdings Corporation, Akishima, Tokyo, Japan). The measurement conditions were as follows: tube voltage, 45 kV; tube current, 200 mA; focal length, 27 mm; and 2θ range, 5–40° (two-dimensional WAXS).

### 2.5. Polarized Optical Microscopy (POM)

Molecular orientation was observed by examining the cross-section of PA11 specimens subjected to high-pressure compression molding at room temperature using an Olympus BX51 polarized optical microscope (Olympus Corporation, Hachioji, Tokyo, Japan).

### 2.6. Tensile Testing

Tensile tests were conducted to investigate the relationship between high-pressure compression molding conditions (temperature and load) and the tensile strength and elongation at break, using a universal testing machine (Strograph VG, Toyo Seiki Seisakusho, Ltd., Kita-ku, Tokyo, Japan). The tests were carried out at a crosshead speed of 5 mm/min at room temperature. Specimens were prepared by punching out JIS Type 7 dumbbell-shaped samples from the center of the rectangular compressed sample, aligned parallel to its longitudinal direction (i.e., the stretching direction).

## 3. Results

### 3.1. High-Pressure Compression Molding Results

**Figure 2:**
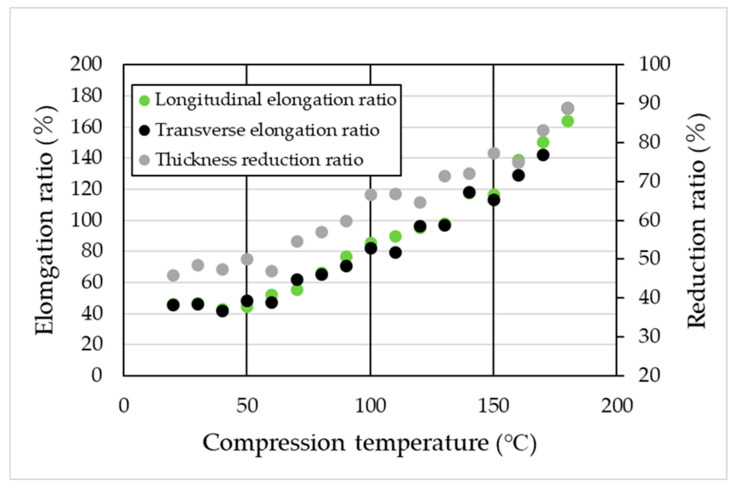
Effect of compression temperature on elongation ratio and thickness reduction ratio.

Figure 2 shows the relationship between compression temperature and both elongation ratio(in the longitudinal and transverse directions) and thickness reduction ratio under a compressive load of 1000 kN. The results indicate that increasing the compression temperature leads to higher elongation ratio and reduction ratio. Notably, the elongation ratio in the longitudinal and transverse directions were nearly identical, suggesting that biaxial stretching occurred during high-pressure thermal compression molding (the maximum strain at 180 °C under a load of 1000 kN was approximately 1.7, with an estimated error of around 10%).

### 3.2. Differential Scanning Calorimetry (DSC) Results

**Figure 3:**
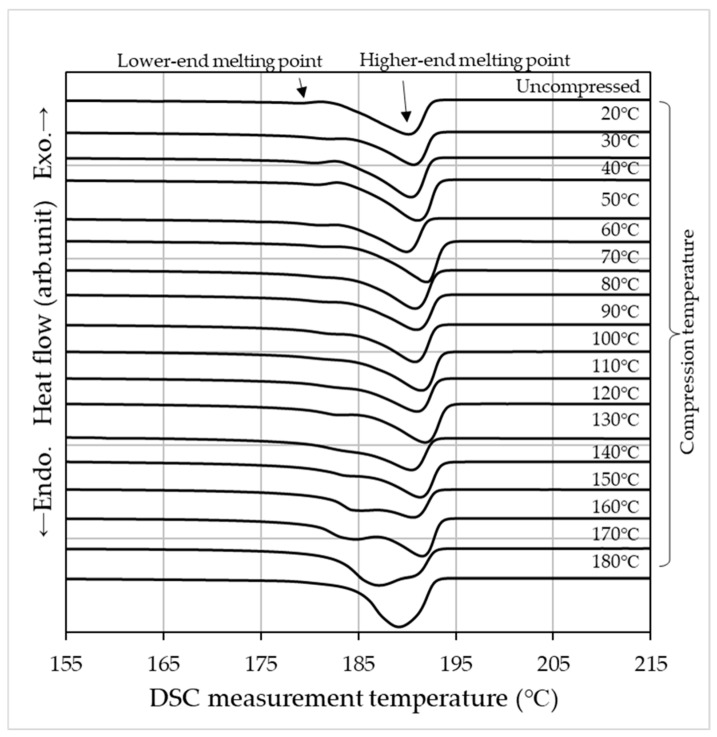
DSC scanning curves of specimens compressed under a 1000 kN load at 10 °C intervals from 20 °C to 180 °C, along with an uncompressed sample.

**Figure 4:**
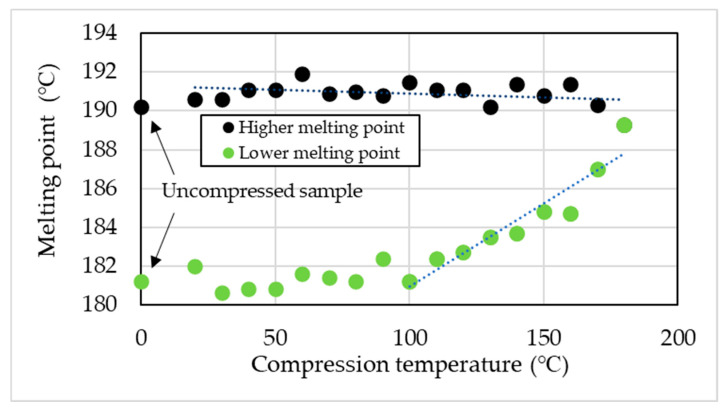
Effect of compression temperature under a 1000 kN load on melting behavior, showing two distinct melting points.

Figure 3 presents the DSC measurement results obtained for specimens compressed and heated under a 1000 kN load at temperatures ranging from 20 °C to 180 °C in 10 °C increments. Note that this DSC measurement is limited to the first heating scan. In the uncompressed PA11 specimen, two distinct endothermic peaks were observed at approximately 181 °C and 190 °C. Previous studies have reported that this dual melting behavior originates from the formation of the α’ phase [13]. This phenomenon reflects the polymorphic nature of PA11, wherein the coexistence of the α’ and α phases becomes pronounced. The crystalline structure is known to vary with cooling rate and applied pressure, thereby influencing the observed melting behavior. When high-pressure thermal compression molding is applied, the lower melting point gradually shifts toward the higher one, and a single melting peak is observed at the compression molding temperature of 180 °C (Figure 4). Based on the DSC results, high-pressure thermal compression molding indicates the possible coexistence of multiple crystalline phases and changes in their relative proportions [11,12,32].

**Figure 5:**
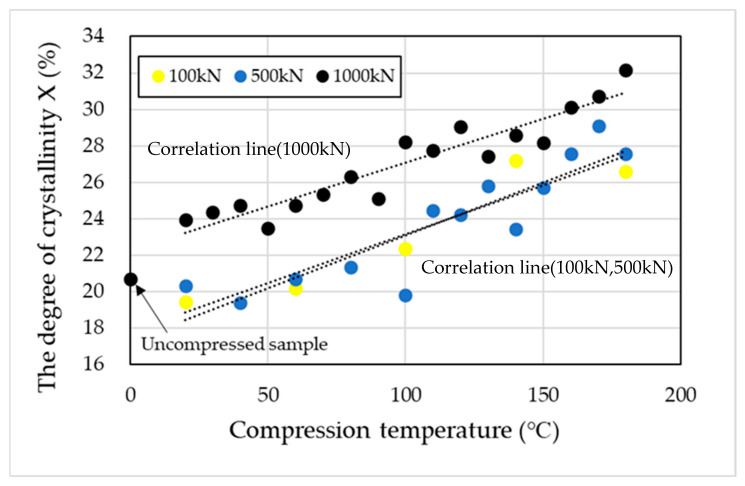
Effect of compression temperature under loads of 100, 500, and 1000 kN on the degree of crystallinity (X).

**Figure 6:**
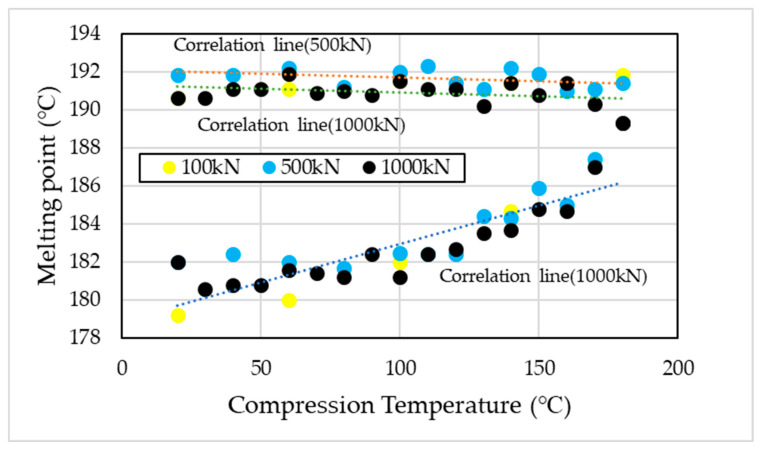
Effect of compression temperature under a 1000 kN load on melting point. There are two melting points.

The degree of crystallinity (X) was calculated using Equation (1) and the fusion enthalpy obtained from the DSC measurements. As the compression molding temperature and compressive load increased, the degree of crystallinity also increased (Figure 5). A similar trend was observed in the melting behavior under high-pressure thermal compression molding at 100 kN and 500 kN, where the lower melting peak shifted toward higher temperatures, consistent with the behavior observed at 1000 kN (Figure 6).

### 3.3. Wide-Angle X-Ray Scattering (WAXS) Results

**Figure 7:**
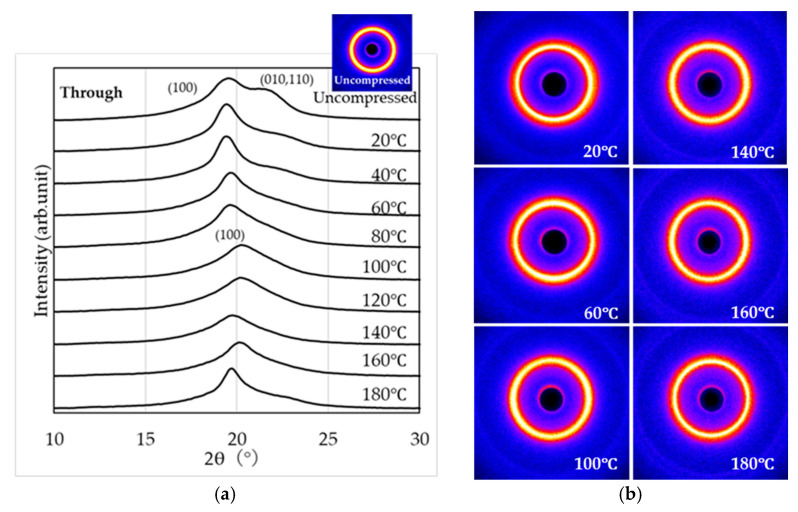
WAXS curves (**a**) and two-dimensional scattering patterns (**b**) obtained in the through-view.

Figure 7 shows the WAXS observation results (through-view) obtained by irradiating X-rays perpendicular to the compressed surface of the specimen subjected to a 1000 kN load. From this viewing direction, no molecular orientation is observed. Diffraction peaks are detected for the (100) and (010, 110) planes, suggesting the formation of the α, α’, and δ’ phases. In the temperature range of 100 °C to 140 °C, a broad peak is observed for the (100) plane.

**Figure 8:**
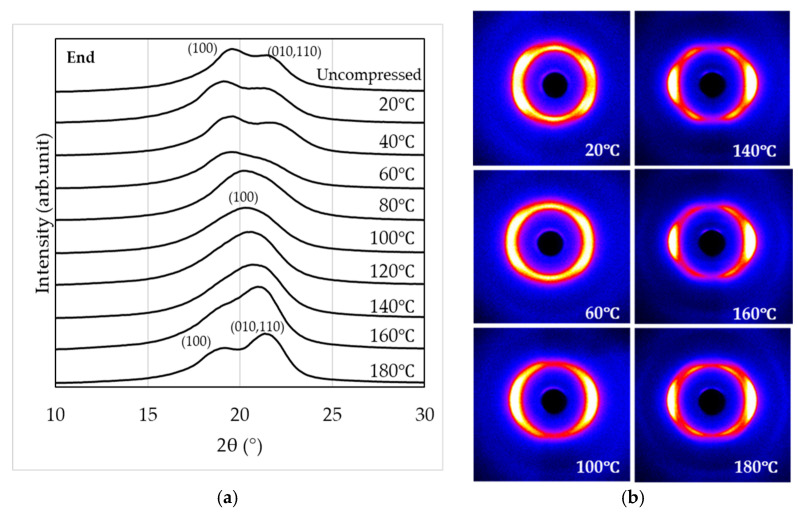
WAXS curves (**a**) and two-dimensional scattering patterns (**b**) obtained in the end-view.

**Figure 9:**
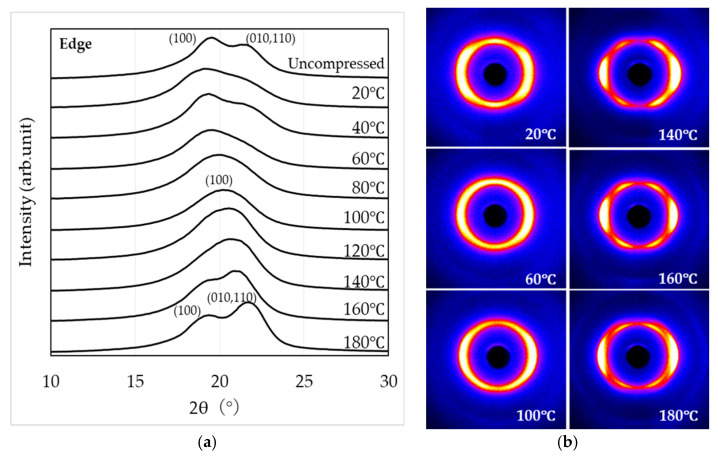
WAXS curves (**a**) and two-dimensional scattering patterns (**b**) obtained in the edge view.

Figure 8 and Figure 9 show the WAXS observation results obtained by irradiating X-rays parallel to the compressed surface of the sample subjected to a 1000 kN load (end-view and edge view, respectively).

From the end-view observation in Figure 8, two peaks corresponding to the (100) and (010, 110) planes appear in the temperature ranges of 20–60 °C and 160–180 °C, while a single peak corresponding to the (100) plane is observed between 80 °C and 140 °C. Furthermore, the two-dimensional WAXS patterns indicate that molecular orientation along the compression plane becomes more pronounced with increasing temperature. The strongest orientation is observed between 140 °C and 160 °C, whereas at 180 °C the degree of orientation appears to be slightly reduced. From the edge view observation in Figure 9, similar to Figure 8, two peaks corresponding to the (100) and (010, 110) planes appear in the temperature ranges of 20 –60 °C and 160 –180 °C, while a single peak corresponding to the (100) plane is observed between 80 °C and 140 °C. In addition, the two-dimensional WAXS patterns reveal that molecular orientation along the compression plane becomes more pronounced as the temperature increases. The strongest orientation is observed between 140 °C and 160 °C, whereas at 180 °C, the degree of orientation appears to be slightly reduced.

**Figure 10:**
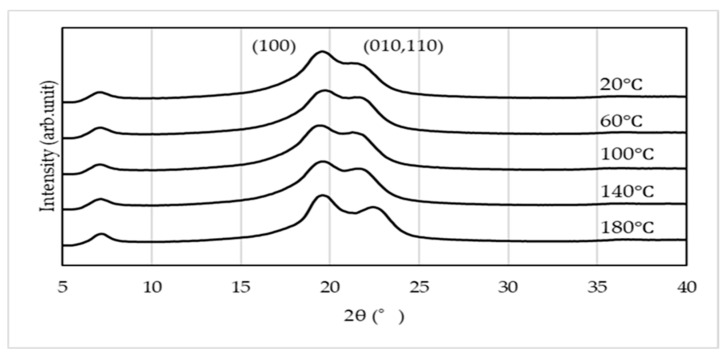
WAXS curves of specimens thermally treated without compression molding, obtained in the through-view.

The WAXS curves of the specimens subjected to thermal treatment without compression molding are presented in Figure 10. These results, obtained in the through-view, provide insight into the crystalline characteristics of PA11 under purely thermal conditions. In specimens subjected only to heat treatment, the α and α’ phases are observed at room temperature, whereas the δ’ phase is absent. PA11 undergoes a Brill transition as a reversible phase transformation, in which the α’ phase at room temperature converts to the δ’ phase at elevated temperatures. However, in specimens processed by high-pressure thermal compression molding, peaks corresponding to the δ’/δ phases are detected even at room temperature. This indicates that the application of pressure suppresses the Brill transition and stabilizes the δ’, δ phases [11,12,32].

**Figure 11:**
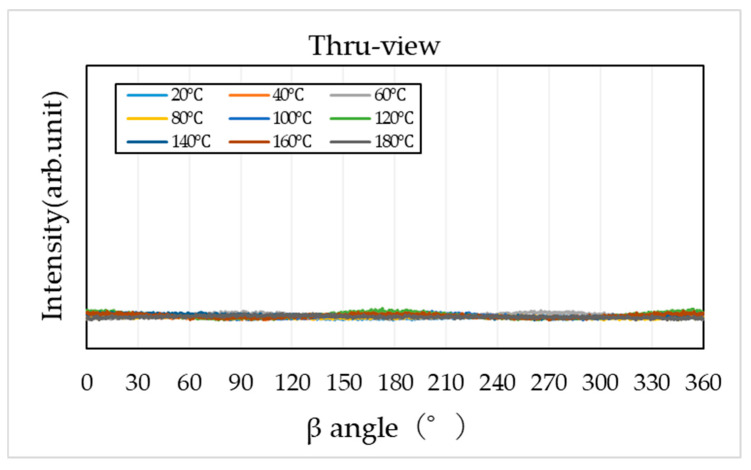
Azimuthal intensity (β–I) profile obtained from WAXS in the through-view.

**Figure 12:**
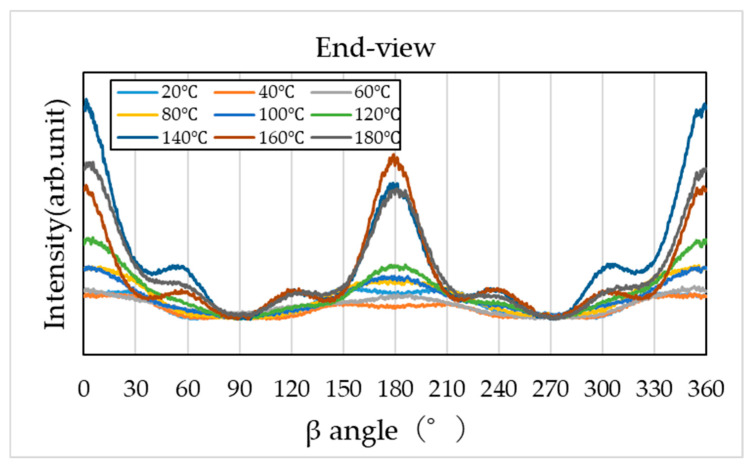
Azimuthal intensity (β–I) profile obtained from WAXS in the end-view.

**Figure 13:**
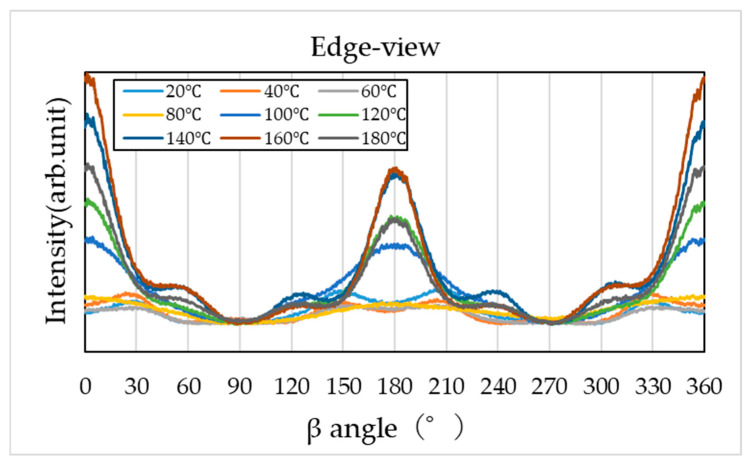
Azimuthal intensity (β–I) profile obtained from WAXS in the edge view.

To evaluate molecular orientation, the diffraction region corresponding to specific crystal planes (2θ = 17–24°) was extracted from the two-dimensional diffraction patterns and converted into β–I profiles along the azimuthal direction (0–360°). The results are shown in Figure 11, Figure 12 and Figure 13.

The β–I profile obtained from the through-view shows minimal intensity variation along the azimuthal direction, indicating the absence of molecular orientation (Figure 11).

However, the β–I profiles obtained from the end view and edge view (Figure 12 and Figure 13) exhibit clear intensity variations along the azimuthal direction, indicating strong molecular orientation, particularly along the compression plane (0°, 180°, and 360°). The degree of orientation increases with higher compression temperatures, becoming most pronounced at 140–160 °C, but slightly relaxes at 180 °C.

### 3.4. Polarized Optical Microscopy (POM) Observations

**Figure 14:**
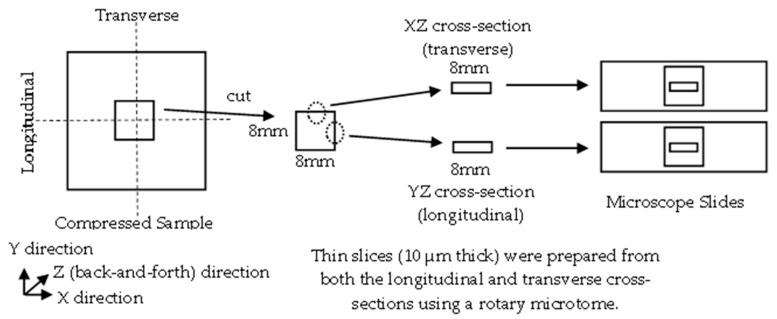
The scheme of sample preparation.

An approximately 8 mm × 8 mm section was excised from the center of the pressed sample using a utility knife. Thin slices with a thickness of 10 μm were then prepared in both the longitudinal and transverse directions using a rotary microtome (RX-860, Yamato Kohoki Industrial Inc., Saitama, Japan). These sections were mounted between a glass slide and a cover slip to produce microscope slides, which were subsequently used for POM analysis (Figure 14).

**Figure 15:**
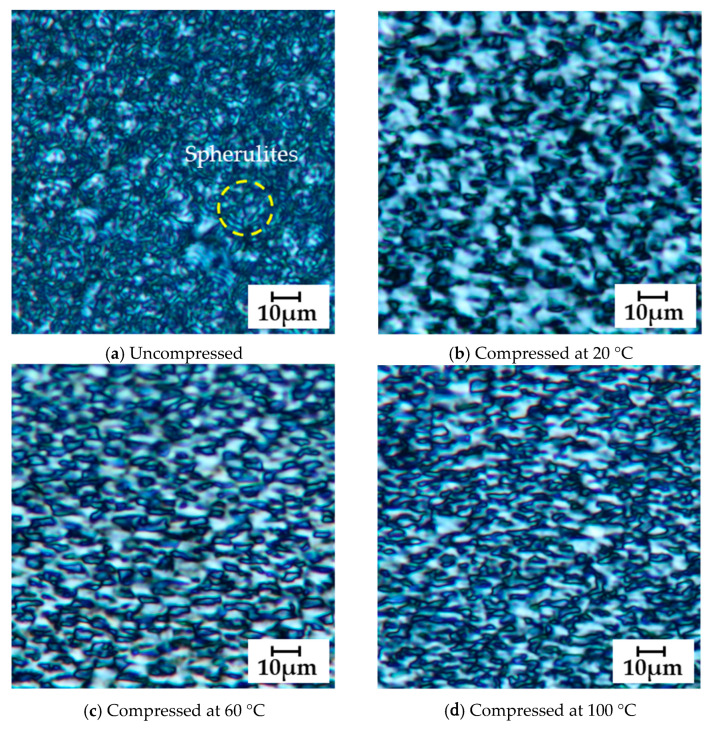

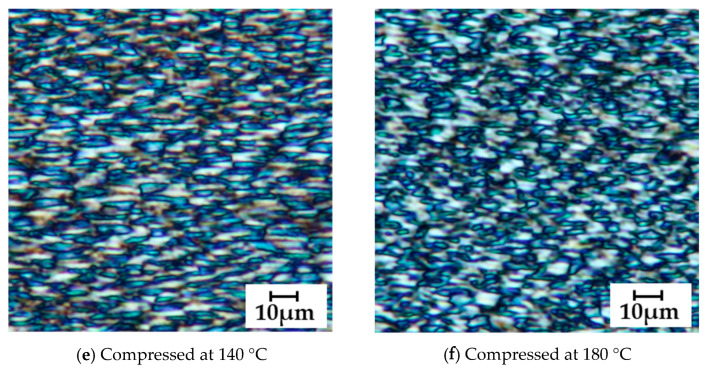
(**a**) shows a polarized optical micrograph of the uncompressed specimen. (**b**–**f**) show polarized optical micrographs of specimens compressed at various temperatures, with compression applied vertically from the top and bottom. Each image was taken at a magnification of 50×. Figure 15 presents the central region observed in the XZ direction, which is representative due to negligible differences between the XZ and YZ views.

Figure 15a–f shows polarized optical micrographs of cross-sections of PA11 specimens, including an uncompressed sample and samples subjected to high-pressure compression molding at various temperatures under a 1000 kN load, observed using an Olympus BX51 polarized optical microscope (Olympus Corporation, Hachioji, Tokyo, Japan).

In the uncompressed specimen (Figure 15a), spherulitic structures are observed; however, these structures disappear after compression. As the applied pressure increases (Figure 15b–e), the microstructures gradually become oriented in the direction perpendicular to the compression axis, that is, the stretching direction. At 140 °C, elongated microstructures with a length of approximately 10 μm are aligned horizontally, exhibiting pronounced orientation.

In contrast, at 180 °C, granular microstructures appear between the elongated ones, and the orientation tends to be slightly relaxed (Figure 15f).

### 3.5. Tensile Testing Results

**Figure 16:**
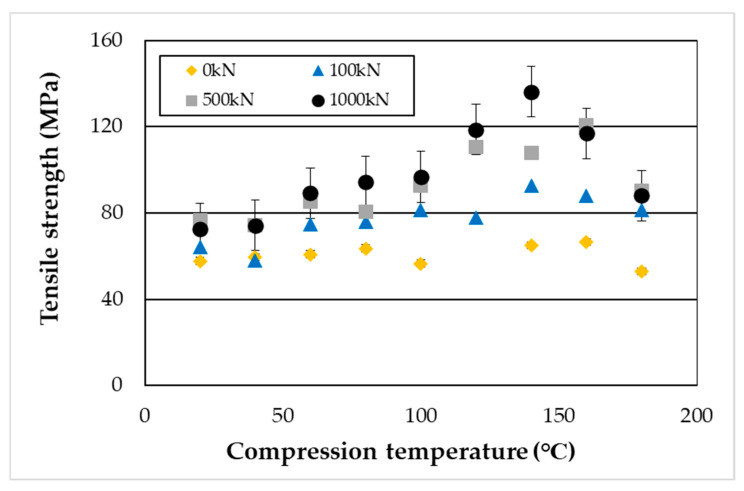
Tensile fracture strength of specimens compressed under loads of 100, 500, and 1000 kN.

**Figure 17:**
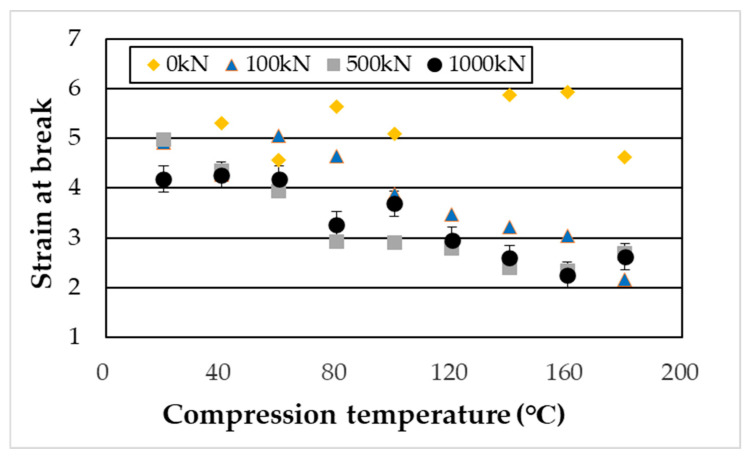
Tensile fracture strain of specimens compressed under loads of 100, 500, and 1000 kN.

The results of the tensile testing (seven repetitions were performed) are shown in Figure 16 and Figure 17. Tensile strength reached its maximum when high-pressure thermal compression molding was applied at 140–160 °C and was further enhanced with increasing compression load. Furthermore, under compressive conditions, the tensile fracture strain tended to decrease with increasing heating temperature.

## 4. Discussion

The α’ phase of PA11 exhibits two distinct melting peaks at approximately 181 °C and 190 °C, whereas the α and δ’ phases display a single melting peak, with the α phase melting at a lower temperature [13]. In this study, two melting peaks were observed in the uncompressed specimens, indicating the formation of the α’ phase. Upon applying high-pressure thermal compression molding, the lower-temperature melting peak shifted toward the higher-temperature side (Figure 3, Figure 4 and Figure 6). Ultimately, under the condition of 180 °C and 1000 kN, the two melting peaks merged into a single peak at 189 °C, approximately 2 °C lower than the original higher melting point. This suggests a phase transition (rearrangement) from the α’ phase to the more ordered α phase.

As shown in Figure 5, the degree of crystallinity increased with both compression temperature and pressure, exhibiting clear temperature and pressure dependence, with a maximum at 180 °C. In isotropic field crystallization (i.e., quiescent crystallization), polymer chains adopt a random coil conformation, forming an isotropic melt from which spherulites are generated [46,47,48]. In fact, spherulitic structures were observed in the POM images of uncompressed specimens, confirming that quiescent crystallization had occurred.

In contrast, when crystallization is induced under anisotropic fields such as elongational or shear flow (i.e., flow-induced crystallization), it is well known that the crystallization rate increases and highly oriented crystalline structures are formed [46,47,48]. In this study, the results of crystallinity and POM images after high-pressure thermal compression molding suggest that flow-induced crystallization accompanied by molecular orientation occurred, with this effect becoming more pronounced under higher compression loads. WAXS measurements (Figure 7, Figure 8 and Figure 9) revealed that the structures formed by high-pressure thermal compression molding were not uniform, but reflected differences in the relative fractions of the α, α’, and δ’ phases. Furthermore, as shown in Figure 10, when only thermal treatment was applied, diffraction peaks corresponding to the (100), (010, 110) planes were observed at all compression temperatures. These peaks indicate the presence of α’ and α phases rather than δ’ or δ phases. This suggests that although δ’ and δ phases may form temporarily at elevated temperatures, they transform into α’ and α phases during cooling to room temperature via Brill transition. On the other hand, under high-pressure thermal compression molding, δ’ and δ phases were retained within the compression temperature range of 100–140 °C. The suppression of Brill transition under these conditions allowed the crystalline structures formed during pressing to be preserved even after cooling. However, a more detailed analysis of the crystalline phases requires WAXS measurements during heating and cooling, which will be addressed in future studies.

As shown in Figure 16, the tensile fracture strength of PA11 exhibited clear dependence on both temperature and pressure, reaching a maximum under the condition of 140 °C and 1000 kN—approximately 2.4 times higher than that of the uncompressed specimen. As illustrated in Figure 17, the tensile fracture strain decreased with increasing compression temperature and load. This reduction is attributed to the increased crystallinity, which enhances stiffness and reduces deformability. Moreover, β-I WAXS profiles and POM images (Figure 11, Figure 12, Figure 13 and Figure 15) revealed that molecular orientation was highest at compression temperatures of 140–160 °C. At 180 °C, however, the oriented structures underwent rearrangement, crystallization progressed, and orientation was relaxed. In the 140–160 °C range, the effects of both crystalline phase transitions (δ’, α, and α’ phases) and flow-induced orientation and crystallization appeared to be present.

In contrast, at 180 °C, although some influence of the flow field remained, isothermal (quiescent) crystallization became dominant. As a result, the elongated structures (it is presumed to be a deformed spherulite) observed in the stretching direction at 140 °C transformed into fine granular structures at 180 °C. Additionally, the elongated structures in the transverse (stretching) direction expanded into the longitudinal (compression) direction, indicating structural rearrangement and relaxation of orientation. These findings suggest that high-pressure thermal compression molding of PA11 induces complex interactions among Brill transition, flow-induced molecular orientation and crystallization, and quiescent crystallization with structural rearrangement, all of which are strongly influenced by temperature and pressure conditions. In particular, the maximum tensile fracture strength observed at 140 °C and 1000 kN is suggested to be attributed to the combined effects of crystalline phase transformation (from α’ and α to δ’ phase) via Brill transition and flow-induced molecular orientation and crystallization in the δ’ phase. At higher molding temperatures, although the elongation and reduction ratios of the specimens increased and crystallinity improved due to the formation of the α phase and isothermal crystallization, the degree of molecular orientation decreased, resulting in a reduction in tensile fracture strength.

However, this discussion is based solely on observations made after compression molding, and further in-depth analysis conducted during heating or under compression molding conditions will be required in future studies.

These findings suggest that controlling temperature and pressure can alter the molecular orientation and crystallization behavior, thereby enhancing mechanical properties of PA11. This, in turn, may lead to improved product performance and open up possibilities for new applications, contributing to the broader adoption of such biomass-derived polymers (sustainable materials) and to environmental sustainability.

## 5. Conclusions

This study demonstrated that high-pressure thermal compression molding significantly modifies the crystalline structure and mechanical properties of PA11, specifically the grade Rilsan^®^ BMN O TLD—a fully bio-based resin derived from castor oil and manufactured by Arkema. Notably, the tensile fracture strength reached a maximum—approximately 2.4 times higher than that of the uncompressed sample—under the experimental conditions of 140 °C and 1000 kN. This enhancement is primarily attributed to the molecular orientation and crystallization of the δ’ phase, with significant contributions from the suppression of the Brill transition and flow-induced crystallization. In contrast, at 180 °C, although the degree of crystallinity increased, molecular orientation decreased, resulting in reduced tensile strength. These findings indicate that the mechanical properties of PA11 are governed by a complex interplay among phase transitions, molecular orientation, and crystallization, all of which are strongly influenced by temperature and pressure conditions.

The results suggest that precise control of temperature and pressure during processing can markedly enhance the mechanical strength of PA11. Future research will aim to elucidate the effects of high-pressure thermal compression molding on the structure and properties of PA11-based composites incorporating bio-based fillers derived from renewable resources, with the goal of contributing to the development of high-performance, environmentally sustainable materials.

## Data Availability

The original contributions presented in this study are included in the article. Further inquiries can be directed to the corresponding authors.
